# Effects on Plant Growth and Reproduction of a Peach R2R3-MYB Transcription Factor Overexpressed in Tobacco

**DOI:** 10.3389/fpls.2019.01143

**Published:** 2019-10-18

**Authors:** Md Abdur Rahim, Francesca Resentini, Francesca Dalla Vecchia, Livio Trainotti

**Affiliations:** ^1^Department of Biology, University of Padova, Padova, Italy; ^2^Orto Botanico, University of Padova, Padova, Italy

**Keywords:** anthocyanin, epidermis, flower, gametophyte, *Nicotiana tabacum*, trichome, R2R3-MYB, transcription factor

## Abstract

In plants, anthocyanin production is controlled by MYB and bHLH transcription factors. In peach, among the members of these families, *MYB10.1* and *bHLH3* have been shown to be the most important genes for production of these pigments during fruit ripening. Anthocyanins are valuable molecules, and the overexpression of regulatory genes in annual fast-growing plants has been explored for their biotechnological production. The overexpression of peach *MYB10.1* in tobacco plants induced anthocyanin pigmentation, which was particularly strong in the reproductive parts. Pigment production was the result of an up-regulation of the expression level of key genes of the flavonoid biosynthetic pathway, such as *NtCHS*, *NtCHI*, *NtF3H*, *NtDFR*, *NtANS*, and *NtUFGT*, as well as of the proanthocyanidin biosynthetic pathway such as *NtLAR*. Nevertheless, phenotypic alterations in transgenic tobacco lines were not only limited to anthocyanin production. Lines showing a strong phenotype (type I) exhibited irregular leaf shape and size and reduced plant height. Moreover, flowers had reduced length of anther’s filament, nondehiscent anthers, reduced pistil length, aborted nectary glands, and impaired capsule development, but the reproductive parts including androecium, gynoecium, and petals were more pigmented that in wild type. Surprisingly, overexpression of peach *MYB10.1* led to suppression of *NtMYB305*, which is required for floral development and, of one of its target genes, *NECTARIN1* (*NtNCE1*), involved in the nectary gland formation. *MYB10.1* overexpression up-regulated JA biosynthetic (*NtAOS*) and signaling (*NtJAZd*) genes, as well as *1-aminocyclopropane-1-carboxylate oxidase* (*NtACO*) in flowers. The alteration of these hormonal pathways might be among the causes of the observed floral abnormalities with defects in both male and female gametophyte development. In particular, approximately only 30% of pollen grains of type I lines were viable, while during megaspore formation, there was a block during FG1 (St3-II). This block seemed to be associated to an excessive accumulation of callose. It can be concluded that the overexpression of peach *MYB10.1* in tobacco not only regulates flavonoid biosynthesis (anthocyanin and proanthocyanidin) in the reproductive parts but also plays a role in other processes such as vegetative and reproductive development.

## Introduction

Peach [*Prunus persica* (L.) Batsch] is one of the most economically important fruit crops that belongs to the Rosaceae family. The world peach and nectarine production is more than 24.97 million tons (http://www.fao.org/faostat/en/#data/QC, accessed on 29 July 2018). The largest world producer of peaches and nectarines is China followed by Italy and the United States. The fruit color is one of the major quality traits in peach. The visible red coloration of peaches is mainly due to the accumulation of anthocyanin pigments. During ripening, many fruits accumulate different types of bioactive chemicals, including anthocyanins that give protection to human health against cancer and cardiovascular, neurodegenerative, and other chronic diseases (Rao and Rao, 2007; Butelli et al., 2008; Singh et al., 2008). Besides, anthocyanins increase antioxidant levels in serum (Mazza et al., 2002), cholesterol distribution (Xia et al., 2007), and restoration of vision disorders (Matsumoto et al., 2003) and help in reducing obesity (Tsuda et al., 2003), as well as protect human red blood cells from oxidative damage (Tedesco et al., 2001). Therefore, red peach is one of the important objectives of the fruit tree breeders for the fresh market acceptability (Ravaglia et al., 2013). The accumulation of anthocyanin pigments is genetically determined by genes coding for enzymes of the anthocyanin biosynthetic pathway and by transcription factors (TFs) controlling their expression (Dixon and Steele, 1999; Petroni and Tonelli, 2011; Jaakola, 2013; Albert et al., 2014). R2R3-MYB TFs are the main regulators of the structural genes encoding enzymes for anthocyanin biosynthetic pathway (Ban et al., 2007; Deluc et al., 2008). In *Arabidopsis*, MYB TFs are classified into three subfamilies based on the presence of number of conserved DNA-binding domains called MYB domains (Stracke et al., 2001; Feller et al., 2011). The MYB TFs are called MYB1R factor (one MYB domain), R2R3-MYB factor (two MYB domains), and MYB3R factor (three MYB domains) based on the number of MYB domain repeats (Stracke et al., 2001). In *Arabidopsis*, there are 137 R2R3-MYB TFs, and some of them regulate flavonoid biosynthesis. In particular, *production of anthocyanin pigment1* (AtPAP1/AtMYB75), AtPAP2 (AtMYB90), AtPAP3 (AtMYB113), and AtPAP4 (AtMYB114) are involved in anthocyanin production (Borevitz et al., 2000; Nesi et al., 2001; Ramsay and Glover, 2005; Stracke et al., 2007; Gonzalez et al., 2008; Heppel et al., 2013), whereas AtMYB123 regulates proanthocyanidin (PA) biosynthesis (Lepiniec et al., 2006). Besides *Arabidopsis*, anthocyanin-promoting MYB TFs are studied in many species, for instance, in tomato (ANT1; (Mathews, 2003)), petunia (AN2; (Quattrocchio et al., 1999)), *Capsicum* (A; (Borovsky et al., 2004)), grape [MYB1a; (Kobayashi et al., 2002)], maize [P; (Grotewold et al., 1991)], sweet potato (MYB1; (Mano et al., 2007)), snapdragon (ROSEA1, ROSEA2 and VENOSA; (Schwinn, 2006)), apple (MYB10, MYB1/MYBA; Takos et al., 2006; (Takos et al., 2006; Ban et al., 2007; Espley et al., 2007; Lin-Wang et al., 2010), strawberry (MYB10, MYB1, and MYB1; (Lin-Wang et al., 2010; Salvatierra et al., 2013)), and also in peach (MYB10, MYB10.1/2/3; (Lin-Wang et al., 2010; Rahim et al., 2014; Ravaglia et al., 2013)).

In the transcriptional regulation of anthocyanin biosynthetic genes, R2R3-MYB TFs do not work alone but in a complex, called MBW, which includes basic helix-loop-helix (bHLH) TFs and WD40 proteins (Xu et al., 2015). Mutants, RNAi, and overexpressing transgenic lines have largely been used to study the function of these genes, also in heterologous systems, as in the case of the overexpression of MYBs from several species in tobacco (Yamagishi et al., 2014; Huang et al., 2016; Li et al., 2016; Liu et al., 2016; Naing et al., 2018). These overexpression studies frequently reported the accumulation of anthocyanins in the host and thus, given the beneficial effects on health and the possible use of those pigments as dyes, suggested their use as tools for biofortification (Butelli et al., 2008) or for wider biotechnological applications (Appelhagen et al., 2018).

In this study, the functional characterization of peach MYB10.1 encoding R2R3-MYB TF was carried out in a heterologous system by stable tobacco transformation. Besides the expected effects on the production of anthocyanins, other processes such as vegetative and reproductive development were changed.

## Materials and methods

### Plant Materials and Growth Conditions

The peach *MYB10.1* was isolated from cv. “Stark red gold” and tobacco (*Nicotiana tabacum*) cv. “Samsung NN” was used to generate transgenic plants. All the cultures were grown in a climate chamber at 22°C under 16-h light/8-h dark condition.

### Gene Isolation and Plasmid Construction

The full-length coding sequence (CDS) of *MYB10.1* (ppa026640m) was amplified by polymerase chain reaction (PCR) from fruit cDNA of peach cv. “Stark red gold” with forward primer (5′-ATGGAGGGCTATAACTTGGGTGT-3′) and reverse primer (5′-TTAATGATTCCAAAAGTCCACGTT-3′) comparing with other known MYB10 TFs from different species. Polymerase chain reaction products were cloned into pCR^®^8/GW/TOPO^®^ vector (Invitrogen), and CDS identity confirmed by sequencing. The cloned DNA was moved into a binary vector modified in house from the pH-TOP (Craft et al., 2005) in order to contain *attR* sites and a *GUS* reporter gene interrupted by a plant intron (Vancanneyt et al., 1990). The CDS in the final expression vector (named *pOp::MYB10.1*) was under the control of a pOp promoter, recognized by the synthetic TF LhG4, cloned on an independent plasmid (named *35S::LhG4*) under the control of the CaMV 35S promoter (construct LhG4 in Craft et al., 2005; see **Supplementary Figure S1** for a schematic map of the two constructs). Finally, binary vectors harboring the desired constructs were transferred into *Agrobacterium tumefaciens* strain LB 3101 as previously described (Rahim et al., 2014).

### Plant Transformation

Tobacco transformation was carried out following a leaf disc cocultivation protocol (Fisher and Guiltinan, 1995). The selected transformants were identified by their ability to root on 200 mg L^−1^ kanamycin and 15 mg L^−1^ hygromycin before being transferred to soil in a greenhouse. Double selection was used to isolate transformants carrying both the *pOp::MYB10.1* and *35S::LhG4* cassettes. Polymerase chain reaction on genomic DNA confirmed the presence of the transgenes in selected clones.

### Enzymatic β-Glucuronidase Assay

The activity of β-glucuronidase (GUS) enzyme was evaluated with the substrate 4-methylumbelliferyl-β-d-glucuronide (MUG). The soluble proteins were extracted from frozen tobacco leaf tissues and homogenized in protein extraction buffer (50 mM NaHPO4 pH 7.0, 10 mM EDTA, 0.1% Triton X-100, and 2 mM β-mercaptoethanol). The GUS enzymatic assay was carried out by incubating protein extract in reaction buffer containing the MUG substrate at 37°C. The reaction was stopped in the solution containing 0.2 M Na_2_CO_3_. The released 4-methylumbelliferone (4-MU) was quantified with a DTX880 Multimode Detector (Beckman Coulter) according to the manufacturer’s instructions. The GUS activity was expressed as nM4-MU released min^−1^ μg^−1^ protein (Jefferson et al., 1987). The protein concentration was measured according to the Bradford method, and data points were normalized by protein quantification (Bradford, 1976).

### RNA Extraction and cDNA Synthesis

Total RNA was extracted from tobacco and peach flowers according to Chang et al. (1993). The yield and purity of RNA were checked by means of UV absorption spectra, whereas RNA integrity was ascertained by agarose gel electrophoresis. cDNA was synthesized from 4 μg of total RNA pretreated with 1.0 unit of RQ1 RNAse-free DNAseI (Promega). Random primers were used in the reaction together with High Capacity cDNA Archive Kit (Life Technologies) following the manufacturer’s instruction.

### Gene Expression Analysis

The expression profiles of *MYB10.1*, *NtAN2*, *NtAN1b*, *NtMYB305*, *NtJAZd*, *NtNEC1*, and the anthocyanin biosynthetic pathway genes (*NtPAL*, *NtCHS*, *NtCHI*, *NtF3H*, *NtDFR*, *NtLAR*, *NtANR1*, *NtANS*, and *NtUFGT*) in flowers of tobacco transgenic and wild-type (WT) plants were compared by means of reverse transcription (RT)–PCR. Primers were designed using Lasergene software package (DNASTAR) (see **Supplementary Table S1**). Primer specificities and amplification efficiencies were checked by PCR using as template a pool of the synthetized cDNAs; thereafter, cycle numbers for each gene were optimized. The *ubiquitin conjugating enzyme E2* (*NtUBC2*) was used as control gene for equal loading. Gel images have been digitized using a Bio-Rad Gel Doc XR system avoiding saturating images.

The expression of peach *MYB10.1*, *MYB10.2*, *MYB10.3*, and *MYB24* was carried out by quantitative real-time PCR using an Applied Biosystems 7500 instrument in different floral parts of peach flower. The reactions were set up in a total volume of 10 μl consisting 5.0 μl of Syber Green PCR Master Mix (Applied Biosystems), 0.05 pmol of each forward and reverse primers and 4.5 μl (1.0 ng/μl dilution) of peach flower cDNA samples as starting template. Polymerase chain reaction conditions were 95°C for 10 min to activate the enzyme followed by 40 cycles of 95°C for 15 s, 60°C for 15 s, and 65°C for 34 s. The obtained *C_T_* values were analyzed using Q-gene software (Muller et al., 2002) considering the means of three independently calculated normalized expression values for each sample. *PpN1* (gene identifier: Prupe.8G137600, formerly ppa009483m, a peach type 2A phosphatase activator TIP41) was used as internal standard for peach.

### Electron Microscopy

Tobacco leaves and flower parts at the same developmental stages were observed under low-pressure conditions by means of environmental scanning electron microscopy (ESEM). The experiment was performed using a FEI Quanta 200 instrument at the CUGAS facilities of University of Padova, Italy. Files of the acquired images were used to measure cell dimensions with the software ImageJ.

### Pollen Viability Assay

The pollen viability was assessed by staining grains with 1% 2,5-diphenyl monotetrazolium bromide (MTT) in 5% sucrose (Norton, 1966; Khatun and Flowers, 1995; Rodriguez-Riano and Dafni, 2000). Tobacco flowers were collected when anthers started to burst; pollen grains were dispersed in 800 µl of MTT solution in 1.5-ml tubes for 10 min followed by centrifugation at 10,000 × *g* for 1 min. Heat-killed (80°C for 2 h) WT tobacco pollen grains were used as negative controls. Ten microliters of grain suspension was placed in a Bürker chamber to count pollen grains under a microscope (Leica DM5000B), equipped with a digital image acquisition system. Pollen grains were considered viable only when they turned deep pink (Wang et al., 2004).

### Pollen *in Situ* Germination and Pistil Observation

Aniline blue staining of pistils was done according to previously described methods (Kho and Baër, 1968; Dumas and Knox, 1983). Briefly, closed flowers (just before anthesis) were emasculated, covered with a paper bag, and left on the plant for additional 7 to 8 h before hand pollination to allow transmitting tract and ovule development. Pistils were pollinated with few pollen grains laid with a small brush on the stigmas and after 48 h were fixed with absolute ethanol/glacial acetic acid (3:1) for 3 h. The fixed pistils were washed three times with dH_2_O for 5 min each, softened in 7.5 N NaOH overnight, and washed in dH_2_O for 1 h each at least three times before staining. Pistils were stained in aniline blue solution (0.1% aniline blue in 0.1 M K_2_HPO4, pH 10.0) for 1 h and observed under a fluorescence microscope (Leica DM5000B, equipped with a digital image acquisition system) using UV (350–400 nm) light.

### Ovule Development Analysis

To analyze the defects in ovule development, flowers at different developmental stages from WT and *MYB10.1* overexpressing lines were fixed overnight at 4°C in 3% glutaraldehyde in 0.1 M sodium cacodylate buffer (pH 6.9) and postfixed at 4°C for 2 h in 1% osmium tetroxide in the same buffer. The specimens were dehydrated in a graded series of ethyl alcohol and propylene oxide and embedded in araldite. Sections were cut using an ultramicrotome (Ultracut S, Reichert-Jung, Wien, Austria). For light microscopy, thin sections (1 µm) stained with toluidine blue (1% basic toluidine and 1% Na tetraborate, 1:1 v/v) were observed using a microscope (DMR 5000 Leica), equipped with a digital image acquisition system.

Ovule development, in flowers at different developmental stages, was followed also in cleared tissues, prepared as reported by Yadegari (1994). Inflorescences were fixed in ethanol:acetic acid 9:1 overnight followed by two washes with 90% and 70% ethanol. Samples were cleared with chloral hydrate/glycerol/water solution (8:1:2) and then dissected under a stereomicroscope and observed using a Zeiss Axiophot D1 microscope equipped with differential interface contrast optics. Images were recorded with an Axiocam MRc5 camera (Zeiss) using the Axiovision program (version 4.1).

In order to detect callose accumulation, whole flowers were fixed and stained as described (Martin, 1959). Handmade thin sections were squashed on a microscope glass and observed using a DMR 5000 Leica microscope, equipped with a digital image acquisition system.

## Results

### Identification and Cloning of the Peach MYB10.1 cDNA

The MYB10.1 cDNA was isolated from peach fruit cv. “Stark red gold.” Analysis of its CDS (720 bp) showed that it encodes an R2R3-MYB TF of 239-amino-acid residues. It has nucleotide sequence homology with anthocyanin-promoting *Arabidopsis* MYB TFs like AtPAP1, AtPAP2, AtPAP3, and AtPAP4 (58.6% and 63.3%, 52.6%, and 57.7% similarity, respectively). Actually, a relatively high similarity (79.2%) was found with MdMYB10 TF (see **Supplementary Table S2**). Constructs used were the same as in Rahim et al. (2014).

### Generation of Transgenic Plants Overexpressing the Peach MYB10.1 Gene

**Figure 1:**
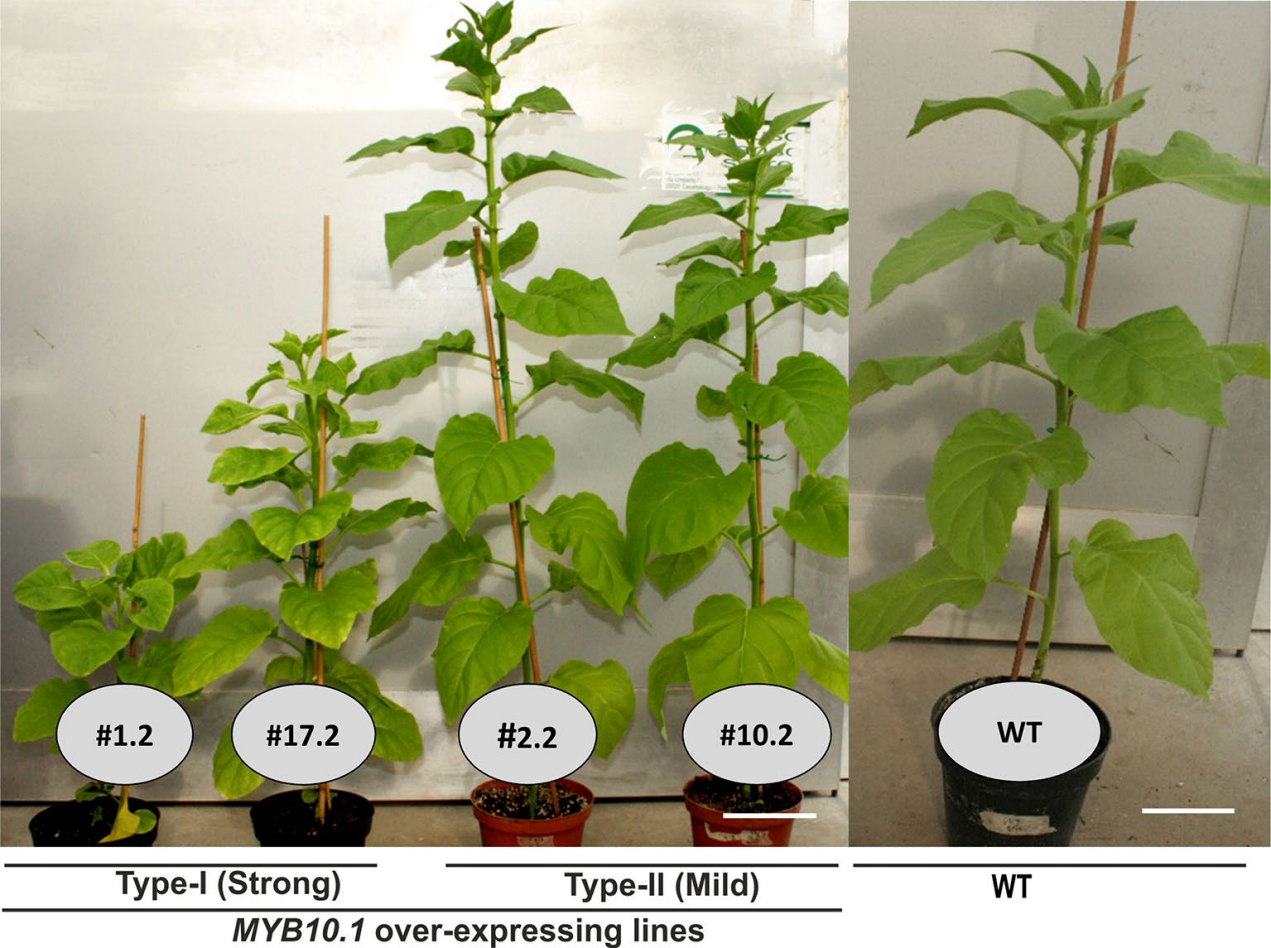
Phenotypes of peach MYB10.1 overexpressing transgenic tobacco lines. Type I, strong phenotype (1.2 and 17.2); type II, mild phenotype (2.2 and 10.2); and WT, wild type.

**Figure 2:**
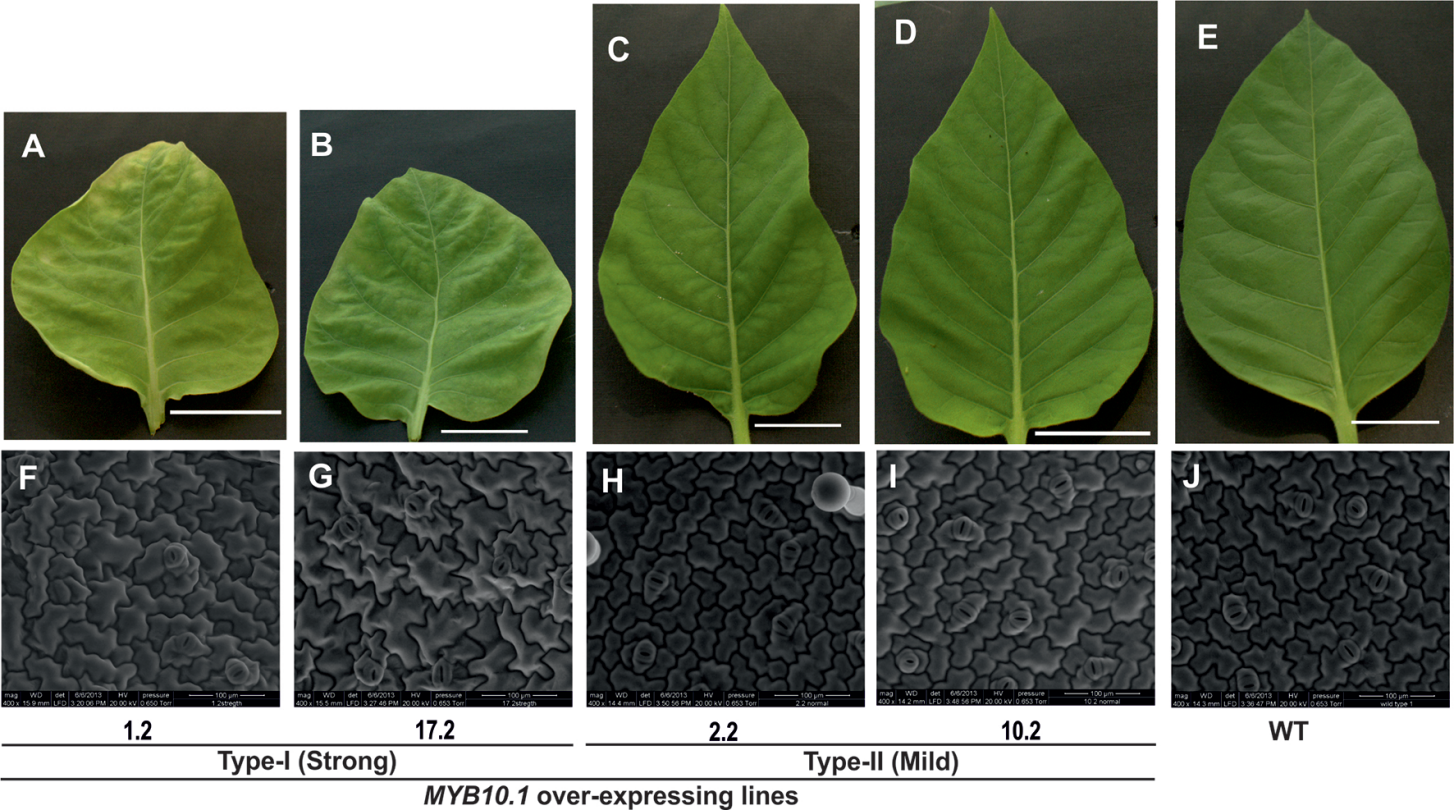
Phenotypic analysis of the upper leaf surface of the transgenic tobacco lines compared to WT. Photographs **(A**–**E)** of leaves with the same age and internode for both transgenic and WT plants. Size bars stand for 5 cm. The images **(F**–**J)** of the upper surfaces of leaves were taken by means of an environmental scanning electron microscope (ESEM). Size bars stand for 100 µm.

**Figure 3:**
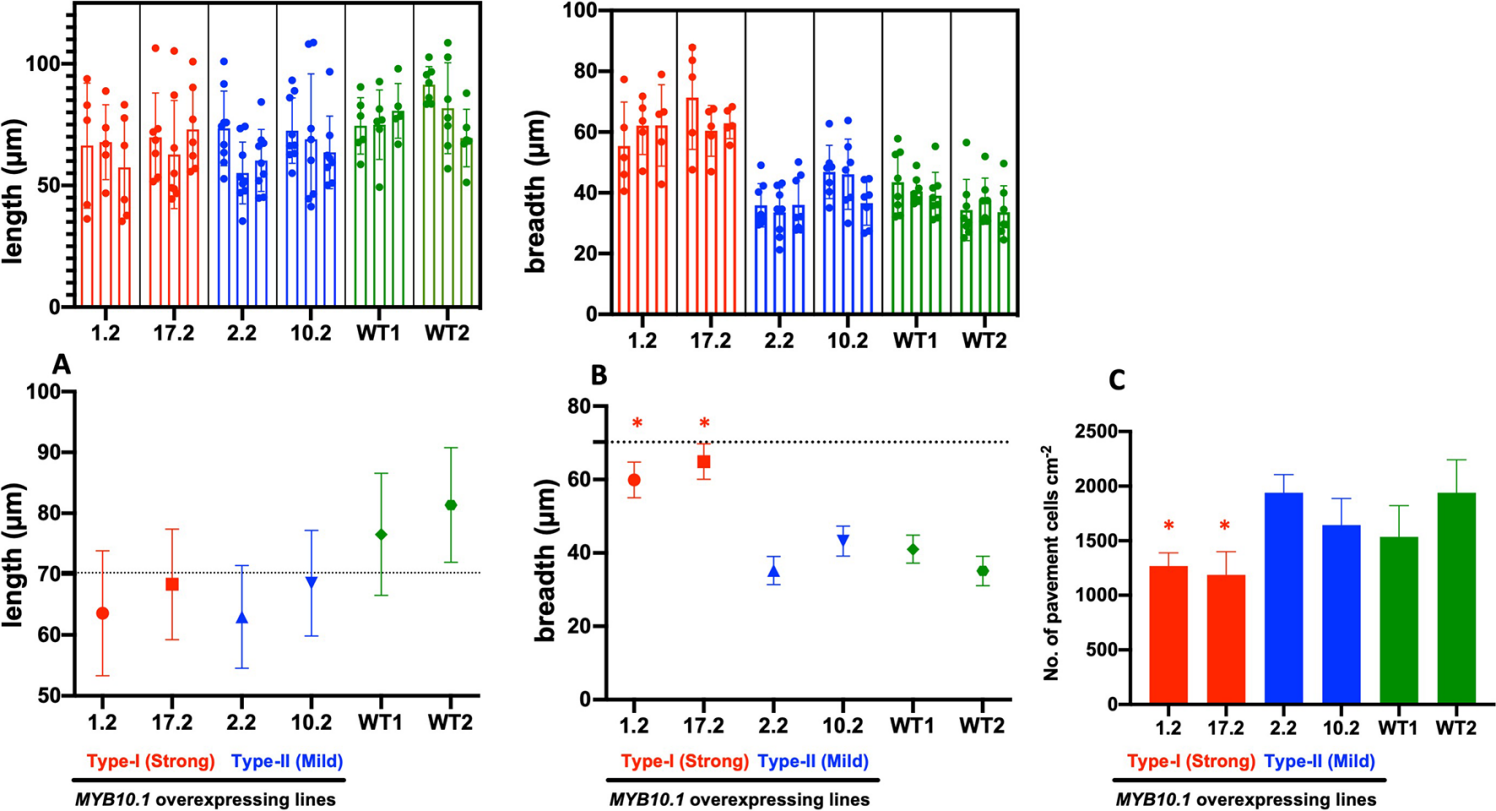
Analysis of the leaf epidermal (adaxial surface) cells of transgenic tobacco lines. Length of the pavement cells **(A)**, breadth of the pavement cells **(B)**, and number of the pavement cells cm−2 **(C)** of the tobacco leaf adaxial epidermis. Length and breadth were measured using the ImageJ software program (Schneider et al., 2012) on photographs taken by ESEM. In the top row of panels **A** and **B**, data are presented for at least five cells (dots) from each plant (column, mean ± SE) of the three used for each independent line (a block for each line); two lines were used for each phenotype (a different color for each phenotype, red for type I and blue for type II). In the bottom row, values are averaged according to the lines (mean ± SD), with the same color code as in the top row. Panel **C** describes the density of pavement cells per surface unit. In this case, data are presented only per single line. Asterisks indicate statistically different values (P < 0.05) from WT with a nested one-way analysis of variance test.

**Figure 4:**
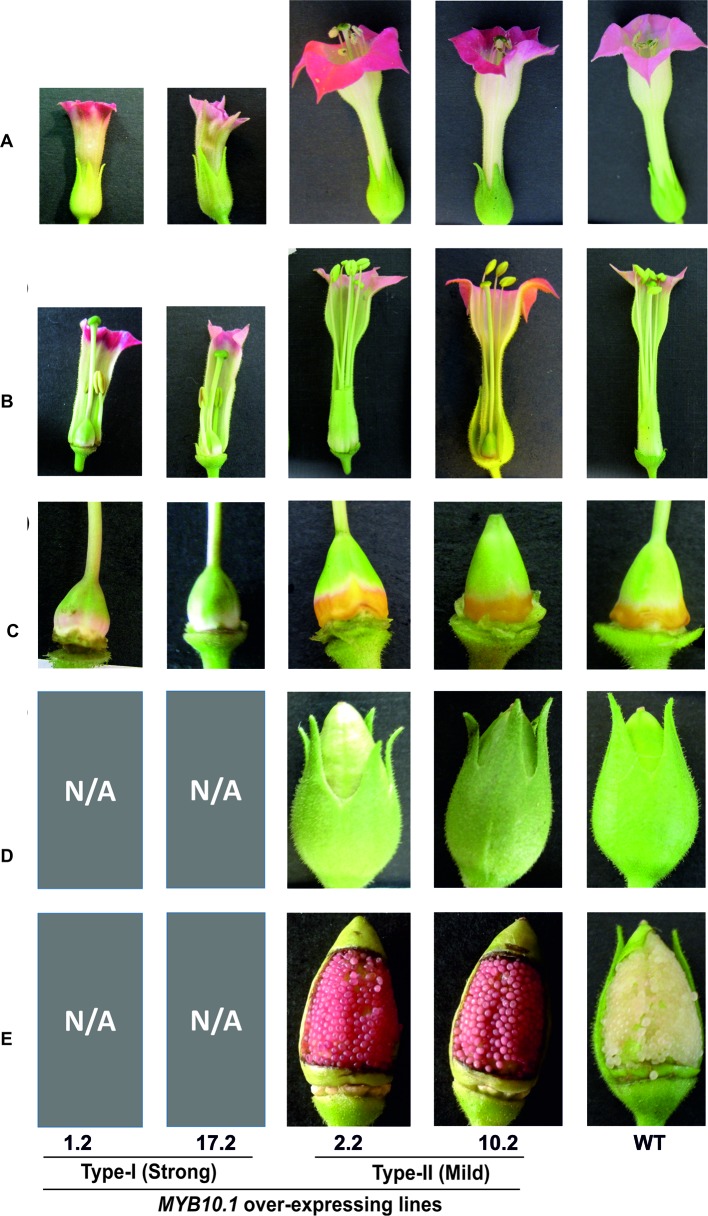
Floral phenotypes of MYB10.1 overexpressing transgenic tobacco lines compared to WT. **(A)** Complete flowers; **(B)** open flowers showing the anther-stigma distance; **(C)** nectary region of the ovary, missing in strong lines; **(D)** green capsules; and **(E)** open capsules showing seed coat pigmentation, reddish in mild phenotypes. N/A indicates the absence of capsules and ovules/seeds in type I transgenic plants.

**Figure 5:**
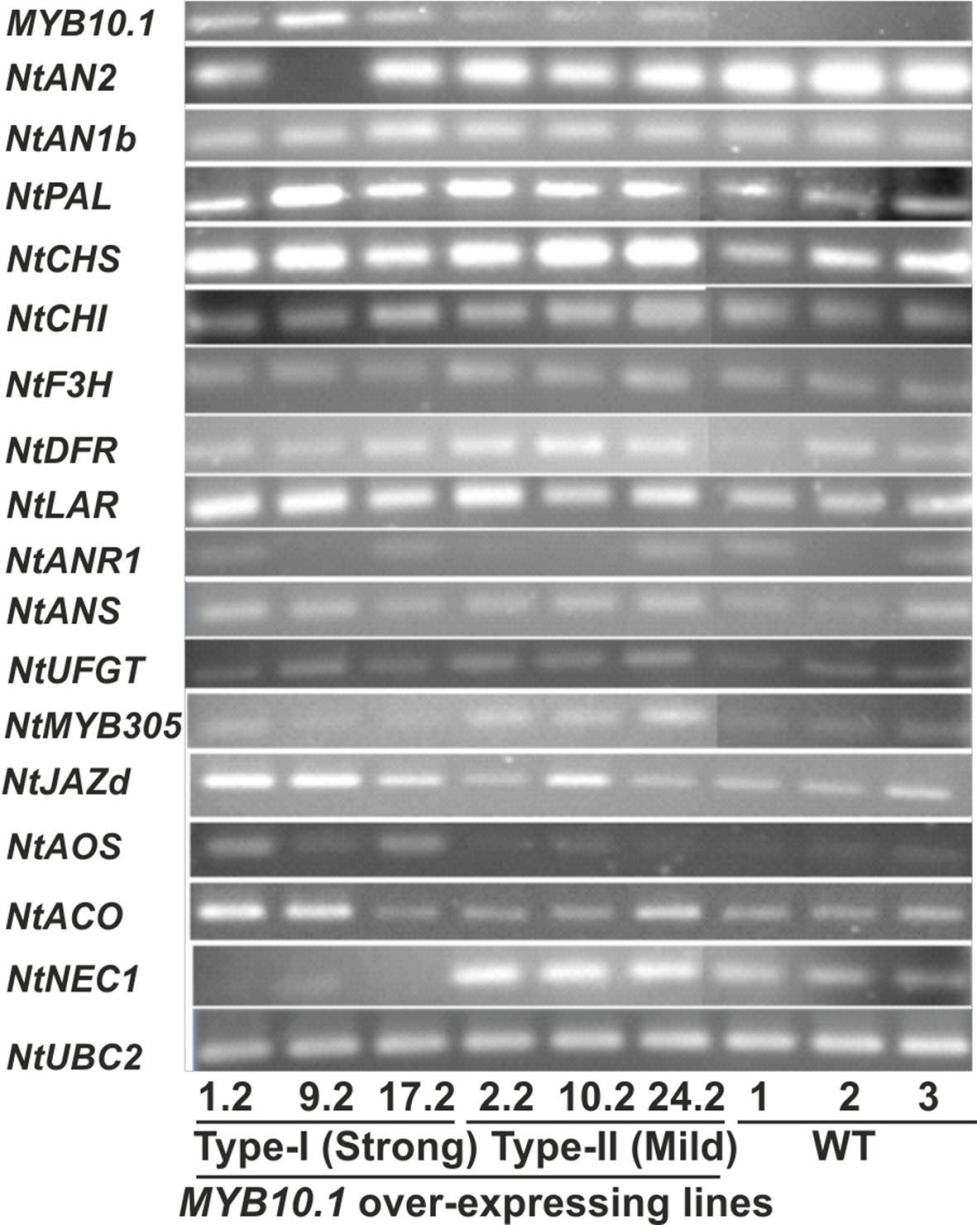
Expression patterns of anthocyanin biosynthetic and flower development genes by RT-PCR in flowers of six independent transgenic and three WT tobacco lines at anthesis. The PCR cycle number was optimized for each gene, and the *Nicotiana tabacum* (Nt) ubiquitin conjugating enzyme E2 encoding gene (NtUBC2, Koyama et al., 2003) was used as a control for equal loading. The genes included in the expression analysis are as follows: MYB10.1, peach MYB10.1 TF gene; NtMYB305, tobacco MYB305 TF gene orthologous of Arabidopsis stamen filament growth related genes AtMYB21, AtMYB24 and AtMYB57 (Cheng et al., 2009); NtPAL, phenylalanine ammonia-lyase; NtCHS, chalcone synthase; NtCHI, chalcone isomerase; NtF3H, flavanone-3-hydroxylase; NtDFR, dihydroflavonol 4-reductase; NtLAR, leucoanthocyanidin reductase; NtANR1, anthocyanindin reductase 1; NtANS, anthocyanidin synthase; UFGT, udp-glycose:flavonoid-3-o-glycosyltransferase; NtJAZd, encodes a jasmonate ZIM-domain protein; NtAOS, allene oxide synthase, NtNEC1, nectarin 1; and NtACO, 1-aminocyclopropane-1-carboxylate oxidase

**Figure 6:**
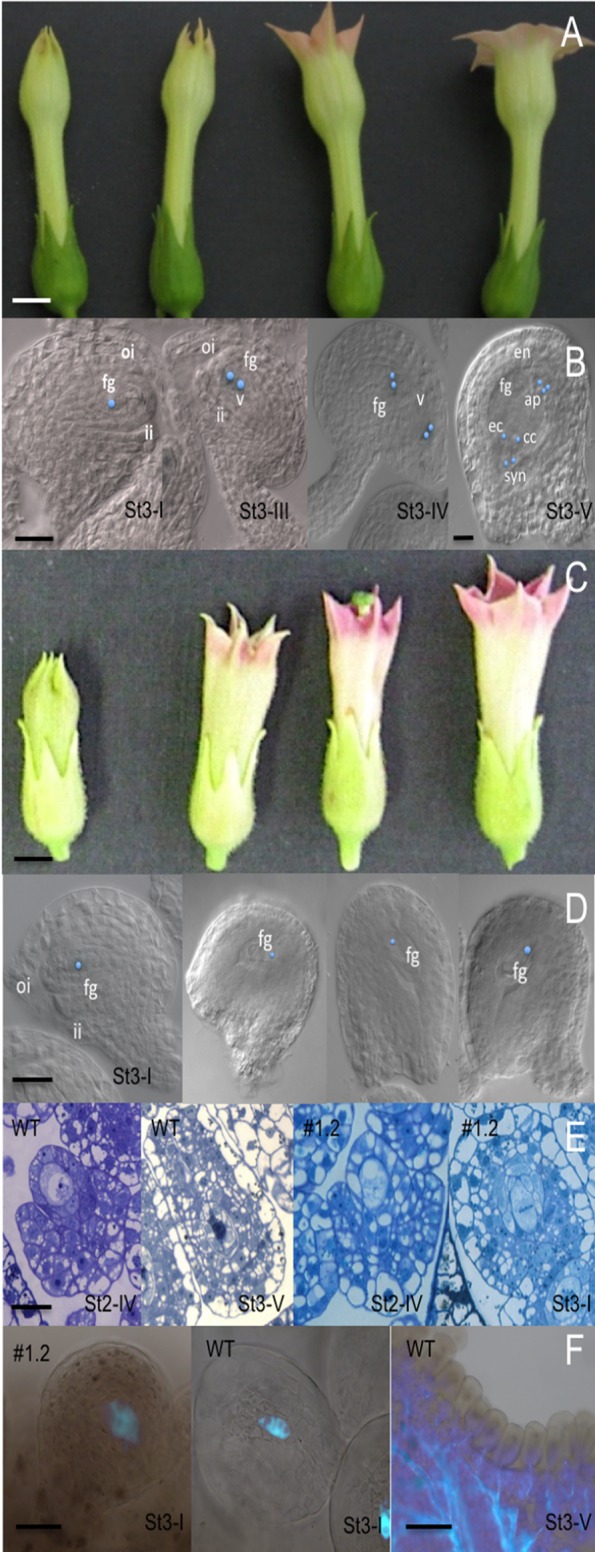
Detailed type I MYB10.1 phenotype during flower development. **(A)** Alteration of floral development in type I compared to **(C)** WT (stages were defined according to Koltunow et al. (1990). Bars = 200 pixel. **(B)** Cleared sections in wild-type developing ovules from stage st3-I to st3-V, the seven-celled embryo sac. **(E)** In ovules of overexpressing lines, the development is blocked to st3-I. **(D)** Thin sections of WT and type I ovules confirm the developmental arrest. **(F)** Aniline blue staining evidenced the persisting deposition of callose in pollinated type I plants, while the callose disappears in pollinated wild-type flowers. Ovules stages are according to Schneitz et al. (1995). ap, antipodal cells; cc, central cell; ec, egg cell; fg, FG; ii, inner integument; oi, outer integument; syn, synergid cells; v, vacuole. Bars are 1 cm in A and B; 50 µm in B, D, E, F St3-I; 200 µm in F St3-V.

**Figure 7:**
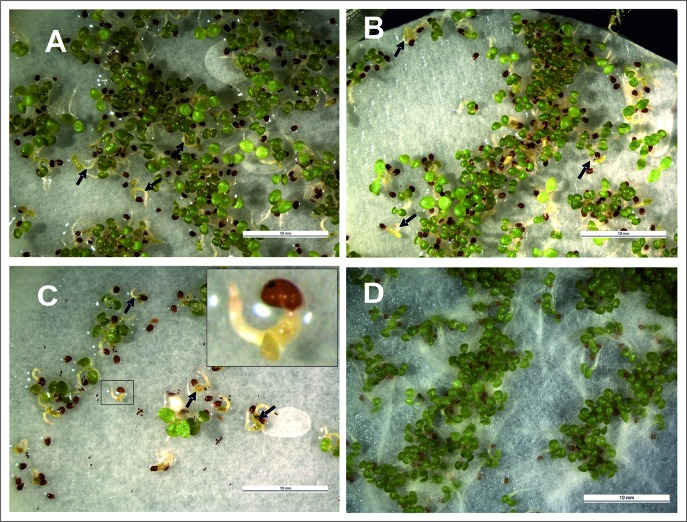
Phenotypes of transgenic **(A**, **B**, and **C**, overexpressing MYB10.1**)** and WT **(D)** tobacco seedlings; **(A)** T_1_ seedlings from the type II 2.2 line (selfed); **(B)** T_1_ seedlings from the type II 10.2 line (selfed); **(C)** F_1_ seedlings from the cross: ♀ 35S::LhG4 x ♂ 35S::MYB10.1. Arrows indicate pigmented cotyledons of transgenic tobacco seedling.

**Figure 8:**
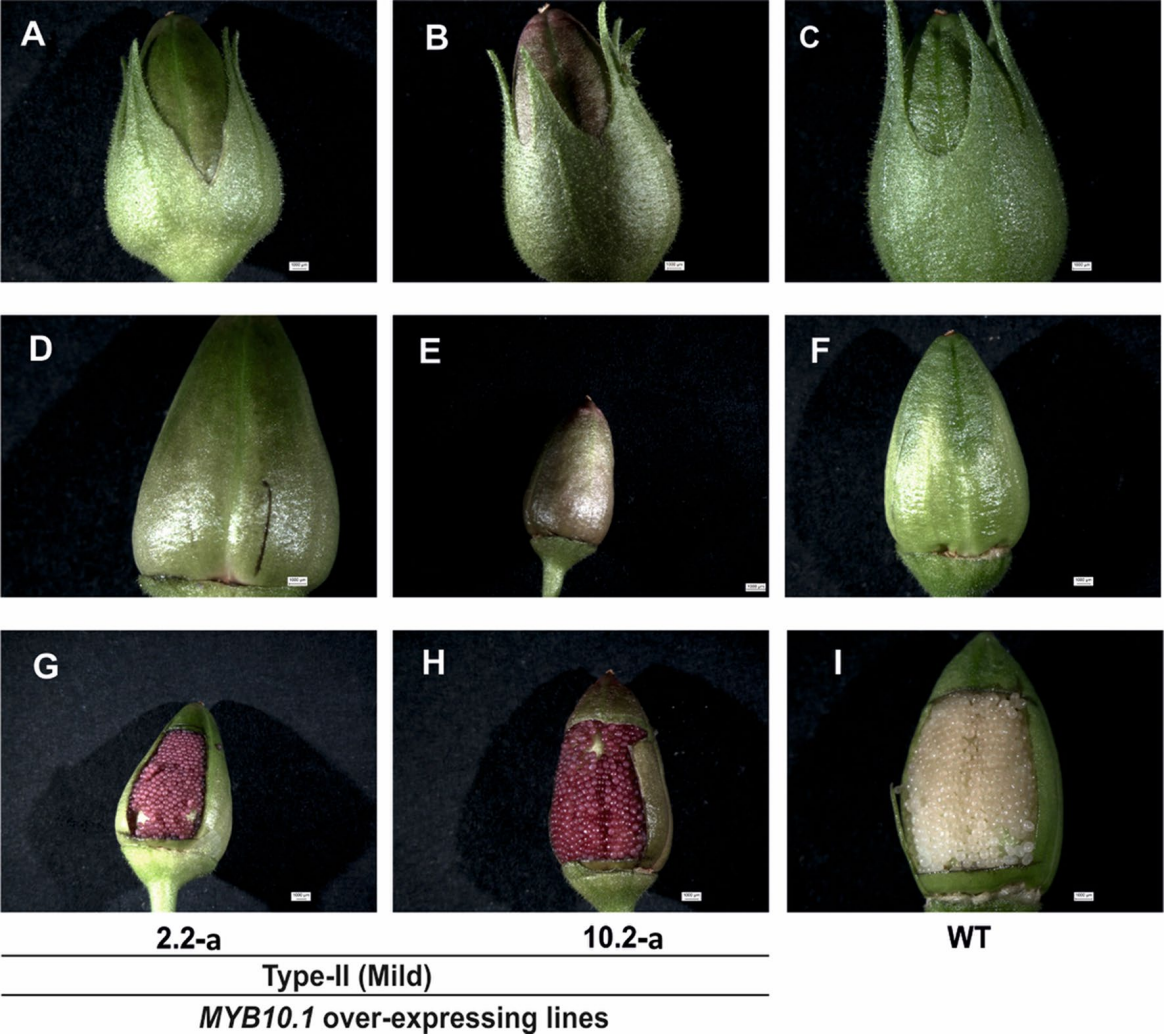
The phenotypes of the capsule and developing seed coat produced by individuals of the T_1_ generation from selfed type II plants, compared to WT. The images **(A**–**C)**, **(D**–**F)**, and **(G**–**I)** indicate capsules with calyx, capsules without calyx, and open capsules showing developing seeds, respectively.

The function of the peach *MYB10.1* gene was analyzed by overexpressing it in the tobacco heterologous system. Thirteen independent lines overexpressing the peach *MYB10.1* gene were obtained from the transformation events, and the presence of the transgene was confirmed by PCR. For the phenotypic and molecular characterization, T_0_ plants were used (**Figures 1**–**6**; **Tables 1** and **2**), but in some cases, also T_1_ and F_1_ (crossing between transgenic plants for *35S::LhG4* and *pOp::MYB10.1* plants) tobacco plants were used for further analysis (**Figures 7** and **8**). The transgenic plants were compared with WT tobacco plants, also propagated by tissue culture, at the same developmental stages.

**Table 1:** Analysis of the length of different floral organs in transgenic tobacco plants overexpressing the peach MYB10.1 compared with WT.

Phenotype	Genotype	Sepal (cm)	Petal (cm)	Androecium (cm)	Gynoecium (cm)
		Mean ± SD	Mean ± SD	Mean ± SD	Mean ± SD
Strong	1.2	1.13 ± 0.06	2.27 ± 0.12	0.99 ± 0.13	1.48 ± 0.03
	17.2	1.2 ± 0.08	2.30 ± 0.18	1.04 ± 0.22	1.70 ± 0.10
Mild	2.2	1.68 ± 0.05	4.55 ± 0.06	4.13 ± 0.10	4.10 ± 0.00
	10.2	1.67 ± 0.04	4.60 ± 0.07	4.19 ± 0.05	4.10 ± 0.08
Control	WT	1.62 ± 0.13	4.94 ± 0.09	4.34 ± 0.05	4.1 ± 0.08

**Table 2:** Percent pollen viability of type I (strong) transgenic tobacco flowers compared to WT.

	Type I (strong)			WT	
	Dehiscent anthers	Nondehiscent anthers	Partially dehiscent anthers	Heat-killed anthers	Non–heat-killed anthers
% Viable pollen	67.20	34.24	34.24	0.00	90.00

### Phenotype of Transgenic Tobacco Plants Overexpressing the Peach MYB10.1 Gene

Several *MYB10.1* overexpressing lines have been obtained, among which individuals had phenotypes ranging from WT-like to severe impairment in growth and development, but none showed evident signs of anthocyanin production. Transgenic plants showing strong defects in vegetative and reproductive characteristics are collectively described as type I, whereas type II includes independent clones showing only minor changes in vegetative and reproductive development when compared with WT tobacco plants (**Figure 1**). Six independent transgenic lines (named 1.2, 5.2, 9.2, 17.2, 19.2, and 20.2) could be assigned to type I and seven (2.2, 8.2, 10.2, 16.2 18.2, 22.2, and 24.2) to type II. Two independent lines (type I: 1.2 and 17.2; type II: 2.2 and 10.2) for each category of phenotype were analyzed further. Since the transgenic plants also contained a GUS reporter gene under the same transcriptional regulatory system (see *Materials and Methods*), GUS activity was assayed. Result showed at least fourfold higher activity in type I compared to type II transgenic plants, while no GUS activity was detected in the WT (see **Supplementary Figure S2**). Thus, GUS activity was, as expected, a good marker for MYB10.1 expression as it was higher in clones showing the strong phenotypes (type I).

At the vegetative level, type I transgenic plants showed reduced plant height (**Figure 1**) and leaf size; furthermore, also leaf shape was altered compared to type II and WT tobacco plants (**Figures 2A–E**). An ESEM analysis of leaf epidermis evidenced that the pavement cells of both types I and II transgenic lines had significant reduction in their lengths compared to WT ones (**Figures 2F–J** and **3A**). Moreover, the breadth of the pavement cells was significantly increased only in type I transgenic lines, while there were no significant differences between type II and WT (**Figure 3B**). In addition, the number of pavement cells per unit area was also significantly reduced in type I transgenic lines, while in type II lines it was similar to WT (**Figure 3C**).

Type I transgenic plants showed abnormalities in floral development, whereas type II transgenic plants had flowers similar to WT (**Figure 4**). Type I transgenic flowers had a huge reduction in the length of the calyx, corolla, androecium, and gynoecium compared to type II and WT (**Figures 4A, B**; **Table 1**). The lengths of the stamen filament were drastically or partially reduced in type I or type II flowers, respectively; as a result, anthers did not reach stigmas, thus preventing autopollination (see **Supplementary Figure S3**). In addition, there was no nectary formation (**Figure 4C**) in type I tobacco flowers. Usually tobacco flowers are pigmented only in petals, and in type II, flowers mimicked WT ones, while in type I transgenic flowers, besides the stronger pigmentation observed in petals, some purple pigmentation was observed also in anthers and ovaries (**Figures 4B, C**). Moreover, in type I transgenic plants, usually anthers did not dehisce; nonetheless, sometimes, at an extremely late stage when flowers were going to drop off, it could be observed that few anthers released some pollen at their tips (see **Supplementary Figure S4A**). At that stage, stigmas had completely lost their ability to allow germination of pollen grains, and thus, all the flowers were fated to drop so that only remnants of inflorescences without capsules remained on the plant (see **Supplementary Figure S4B**). As a consequence of this altered development, no seeds were produced by type I transgenic plants. On the contrary, an increase in petal pigmentation and purple coats in developing seeds were the most striking phenotypic differences observed in type II transgenic plants compared to WT (**Figures 4A–E**).

### Alteration of Gene Expression in Transgenic Tobacco Flowers

The expression of some genes related to anthocyanin biosynthesis and floral development was analyzed by RT-PCR in both the transgenic and the WT tobacco flowers at anthesis. As shown in **Figure 5**, transcript amounts of the peach *MYB10.1* gene were higher in type I transgenic flowers than in type II ones. As expected, *MYB10.1* transcripts were not detected in WT flowers. The expression level of the endogenous *NtAN2* (an R2R3-MYB related to the regulation of anthocyanin biosynthesis in tobacco flower and the ortholog of *MYB10.1*) was low in type I, moderate in type II, and high in WT tobacco flowers. On the other hand, the expression pattern of endogenous *NtAN1b* (encoding bHLH TF) was similar to WT in both types of transgenic flowers.

The transcript abundance of anthocyanin biosynthetic genes was found to be from slightly [chalcone isomerase (NtCHI), flavanone-3-hydroxylase (NtF3H), dihydroflavonol 4-reductase (NtDFR), anthocyanidin synthase (NtANS), and UDP-glucose:flavonoid-3-O-glucosyltransferase (NtUFGT)] to strongly [phenylalanine ammonia-lyase (NtPAL), chalcone synthase (NtCHS)] increased in transgenic plants of both types and induction that seems even stronger in type II than in type I clones (e.g., for CHI, F3H, and DFR genes). The enhanced expression levels of the aforementioned biosynthetic genes correlate with the purple seed coat produced by type II transgenic tobacco plants. A similar expression pattern was also found for leucoanthocyanidin reductase (NtLAR), whereas anthocyanidin reductase 1 (NtANR1) expression was low and not so different from that in WT, meaning that MYB10.1 might also regulate PA biosynthetic genes in tobacco flowers.

As type I transgenic tobacco plants had shown defects in their reproductive organs, other genes encoding MYB TFs related to floral development were also analyzed in this study. In *Arabidopsis*, three MYBs (AtMYB21, AtMYB24, and AtMYB57) are predominantly expressed in flowers (Cheng et al., 2009; Li et al., 2006). As regards tobacco, NtMYB305, besides being the orthologous MYB of the aforementioned *Arabidopsis* genes, controls nectary and flavonoid biosynthetic gene expression in flowers (Liu et al., 2009). Considering these MYBs, a phylogenetic analysis was carried out, and NtMYB305 was confirmed to be orthologous to *Arabidopsis* AtMYB21, AtMYB24, and AtMYB57, whereas only a single peach gene (ppa011751, named MYB24 following the *Arabidopsis* nomenclature) was found in this clade (see **Supplementary Figure S5**). The expression of *NtMYB305*, known to be at maximum between stages 9 and 10 (Liu et al., 2009), was reduced in type I and similar to WT in type II transgenic flowers (**Figure 5**). As the expression of *NtNEC1*, a gene encoding the major nectarine protein, is controlled by *NtMYB305*, it was analyzed in *MYB10.1* overexpressing plants. Not surprisingly, *NtNEC1* was not expressed in type I transgenic flowers, which also had extremely low amounts of *NtMYB305* transcripts, whereas it appeared expressed at similar levels in both type II and WT flowers. These profiles correlate with the nectary glandless phenotype of type I transgenic tobacco flowers (**Figure 4C**).

Jasmonic acid (JA) is required for normal androecium development in *Arabidopsis* acting also through the induction of MYB genes (Mandaokar et al., 2006). As type I plants had phenotypes resembling *Arabidopsis* mutants defective in JA synthesis or signaling (Mandaokar and Browse, 2009), *NtAOS* (*allene oxide synthase*, for JA biosynthesis) and *NtJAZd* (encodes a jasmonate ZIM-domain protein, for JA signaling) were tested for their expression. Both genes were up-regulated in type I transgenic flowers, but had levels similar to WT in type II clones.

The type I transgenic T_0_ plants were unable to set seed either by self-pollination, as occurs in WT flowers, or by manual pollination, even with WT pollen. Ethylene plays a role in megasporogenesis (De Martinis and Mariani, 1999), and thus to investigate whether the hormone had a role in the female reproductive organ development of type I plants, the expression of the *NtACO* gene encoding 1-aminocyclopropane-1-carboxylate oxidase was analyzed. The result showed that the transcript level of *NtACO* was up-regulated in type I transgenic flowers, whereas it was at WT levels in type II plants.

### Functional Analysis of Floral Reproductive Parts of Transgenic Lines

To get insights on the inability to set seed in type I transgenic plants, their male and female reproductive parts were analyzed in more detail. For this purpose, the pollen viability from nondehiscent, dehiscent (only few anthers underwent dehiscence, but at a very late stage, when the flowers had already entered the senescence process), and partially dehiscent anthers was assessed. The pollen viability assay (Wang et al., 2004) showed that approximately 67.2% of pollen grains were viable in dehiscent, 34.24% in the nondehiscent, and 34.38% in the partially dehiscent anthers, while 90% was the viability for the WT pollen (**Table 2**). Therefore, it seems that transgenic pollen grains are less viable than WT but still able to fertilize the ovules, even if the anthers do not open to release grains. To discriminate whether the problem was due to the failure of anther opening or to the pollen maturation process, reciprocal crosses were performed. To check the fertility of the type I transgenic pistils, stigmas of T_0_ flowers were manually pollinated with WT pollen. The fertilization was a total failure, and all the flowers were shed from the plants. On the contrary, when emasculated WT stigmas were hand pollinated with type I pollen from lately dehiscent anthers, there was 85% of successful fertilization (see **Supplementary Table S3**). It has to be noted that most of the seedlings coming from these crosses were GUS positive, thus proving that they originated from transgenic pollen grains (GUS and MYB10.1 are on the same cassette). This indicates that *MYB10.1* somehow affects more the fertility of the female reproductive part than the male one.

To further investigate the effect of *MYB10.1* overexpression on fertility, the female gametophyte development has been monitored in type I and WT plants (**Figures 6A, C**). Three pistils of five independent lines have been analyzed, and a block during ovule formation was noticed. In an analysis of 150 ovules each, in cleared and thin sections, a block has been observed during FG1 corresponding to ovule developmental stage 3-I, confirmed by thin-section investigations (**Figures 6D, E**).

Aniline blue staining evidenced that the deposition of callose at the level of the megaspore persisted during development of ovules in type I plants up to the anthesis stage, while in WT the polymer was readsorbed soon after the tetrad stage (**Figure 6F**).

Type II plants were not considered in these analyses as their flowers were fertile and similar to WT one but for the color of their corollas and seed teguments.

### Phenotype of T_1_ Generation of Transgenic Lines

The T_1_ seeds of type II and F_1_ seeds from crosses between transgenic plant for *35S::LhG4* and *pOp::MYB10.1* plants were sown in Petri dishes on filter paper soaked with water containing kanamycin and hygromycin to allow the growth of only the transgenic plants expressing *MYB10.1*. Some seedlings had a stunted growth, with cotyledons that looked bleached and accumulating pigments and died afterwards (**Figures 7A, B**); on the contrary, no pigments were found in WT cotyledons that grew normally (**Figure 7D**). Also the cotyledons of F_1_ seedlings (♀ *35S::LhG4* × ♂ *pOp::MYB10*.1) showed anthocyanin accumulation and chlorophyll bleaching (**Figure 7C**). After germination on Petri dishes, some of these T_1_ seedlings were transferred to soil and grown in the greenhouse. The presence of the transgene was confirmed by histochemical GUS staining and later by PCR. Several individuals of the T_1_ generation originated from the self-fertilization of type II transgenic plants showed pigment accumulation also in the calyx, capsule, and developing seed coats (**Figure 8**), while no pigment accumulation was observed in the capsule of their previous (T_0_) generation (**Figure 4D**).

## Discussion

There are several reports on both homologous and heterologous overexpression of *MYB* genes like *MdMYBA* in tobacco (Ban et al., 2007), *MdMYB1* in *Arabidopsis* (Takos et al., 2006), *MdMYB10* in apple (Espley et al., 2007), *PyMYB10* in *Arabidopsis* (Feng et al., 2010), *VvMYB5a* (Deluc, 2006) and *VvMYBA1* (Li et al., 2011) in tobacco and grape, *LeANT1* in tobacco and tomato (Mathews, 2003), *IbMYB1a* in *Arabidopsis* (Chu et al., 2013), *IbMYB1* in *Arabidopsis* and sweet potato (Mano et al., 2007), *GMYB10* in tobacco (Elomaa et al., 2003), *AtPAP1* in tobacco, and *Arabidopsis* (Borevitz et al., 2000). Ban et al. (2007) reported that the ectopic expression of *MdMYBA* in tobacco induces the accumulation of anthocyanins in the reproductive tissues. Feng et al. (2010) also demonstrated similar results when *PyMYB10* was overexpressed in *Arabidopsis*. The ectopic expression of *MYB10.1* showed similar anthocyanin accumulation patterns, although always limited to reproductive parts, particularly in petals and developing seeds. Tobacco plants are naturally able to make anthocyanins, but the pathway is only active in the flower, where both MYB (*NtAn2*) and bHLH (*NtAN1a* and *NtAn1b*) genes are expressed (Bai et al., 2011). On the contrary, the anthocyanin biosynthetic pathway is not active in tobacco vegetative parts, as leaves, where *NtAN1a* and *NtAn1b* are not expressed, and thus the overexpression of *MYB10.1* could enhance the anthocyanin pigmentation only in the reproductive parts (flowers and young seeds). The pattern of pigment accumulation is thus dependent on *NtAN1a* and *NtAn1b*, the two bHLH genes that have been shown to participate to the MBW complex in tobacco and whose transcription is presumably not induced by MYB10.1 in vegetative parts, as it was not in flowers (**Figure 5**). The inability of MYB10.1 to induce *NtAN1b* expression makes it different from other R2R3MYBs, as the tobacco *NtAn2*, whose ability to induce the expression of *NtAN1a* and *NtAn1b* provided the necessary elements of the MBW complex to induce anthocyanin synthesis also in leaves (Bai et al., 2011). This inability makes MYB10.1 quite peculiar if compared to other R2R3MYBs of its clade (see **Supplementary Figure S4**). Indeed, Chu et al. (2013) showed that overexpression of *IbMYB1a* gene (coding for sweet potato R2R3-MYB TF) up-regulates structural genes, like *CHI*, *F3H*, and *DFR*, in the anthocyanin biosynthetic pathway in transgenic *Arabidopsis*. Huang et al. (2013a) also demonstrated that overexpression of *EsMYB1* (encoding a R2R3-MYB TF from *Epimedium sagittatum*) up-regulates important flavonoid-related genes in both transgenic tobacco and *Arabidopsis*. Huang et al. (2013b) also found significant up-regulation of anthocyanin biosynthetic genes such as *NtCHS*, *NtCHI*, *NtDFR*, and *NtANS* in transgenic tobacco lines overexpressing *MrMYB1* gene (encoding R2R3-MYB) from Chinese bayberry. Also, the heterologous expression of *MYB10.1* modulates the transcription of most of the structural genes in the anthocyanin biosynthetic pathway, thus leading to purple anthocyanin pigmentation in the reproductive tissues of transgenic tobacco plants, but only in flower. These findings suggest that the overexpression of peach *MYB10.1* up-regulates anthocyanin production in tobacco flower by inducing the transcription of the biosynthetic genes through the interaction with the endogenous WD40 and bHLH coactivators.

In addition to regulation of anthocyanin biosynthesis, which was expected, overexpressing of *MYB10.1* caused other phenotypic variations in transgenic tobacco lines. After analyzing expression pattern of *MYB10.1* in transgenic tobacco plants, it was found that tobacco plants (type I) with higher expression of transgenes were defective with their vegetative and reproductive development. On the contrary, tobacco plants (type II) with moderate expression of transgenes were normal in their vegetative and floral development excluding seed coat pigmentation. The type I transgenic lines exhibited shorter plants and reduced leaf length, but similar leaf breadth as compared to type II and WT. This is probably due to the irregular cell size and shape, which here has been measured only on the most accessible ones, i.e., those of the epidermis. Epidermal cells, indeed, showed reduced length and increased breadth leading to a reduced number of epidermal cells per unit area. This suggests the involvement of *MYB10.1* on the plant cell growth and developments, interfering with a similar regulatory system composed of TTG1(WD40)-EG3/EGL3(bHLH)-GL1(R2R3-MYB) that has been widely studied in *Arabidopsis* epidermal cell differentiation (Grebe, 2012). Although there are reports that sustain that “the trichomes of *Arabidopsis* and *Nicotiana* are merely analogous structures and that the *MYB* genes regulating their differentiation are specific and separate” (Payne et al., 1999) and that the *bHLH* genes have no effect on epidermal cell development in plants belonging to the Asterid division (Serna and Martin, 2006), we cannot exclude that the high expression levels achieved by means of the pOp/LhG4 expression system (Rutherford et al., 2005) of *MYB10.1* had an impact on cell fate determination, reducing their sizes and thus decreasing plant and leaf growth. Altered expression of MYB/bHLH TFs not only induced anthocyanin biosynthesis in tomato leaves, but also led to the up-regulation of *MIXTA-like* and *GLABRA2* (*GL2*) TFs, regulators of epidermal cell patterning and trichome differentiation (Outchkourov et al., 2018). Future molecular investigations on vegetative parts of type I plants and the development of lines with epidermis-specific expression will help to elucidate the genes and pathways affected by *MYB10.1* overexpression in vegetative parts.

The role of MYBs of the PAP/MYB10 clade on flower development was up to now limited to pigment and thus color development, whereas MYBs of other clades have been described to participate to cell patterning and differentiation. It is known that *AtMYB21*, *AtMYB24*, and *AtMYB57* are mainly expressed in the flowers and play a critical role for the reproductive organ development (Shin et al., 2002; Li et al., 2006; Cheng et al., 2009; Dubos et al., 2010; Reeves et al., 2012). Yang et al. (2007) showed that *AtMYB24* was expressed in flowers, specifically in microspores and ovules, and plays an important role in anther development. Overexpression of *AtMYB24* caused pleiotropic phenotypes such as reduced plant height as well as defective anther development. After double mutant (*myb21, myb24*) analysis in *Arabidopsis* (Mandaokar et al., 2006; Reeves et al., 2012), it was shown that *AtMYB21* and *AtMYB24* are involved in floral organ development, such as flower opening, petal expansion, anther filament elongation, anther dehiscence, inhibition of lateral vascular development in unfertilized carpels, and abscission of sepals, petals, and stamens. In tobacco and petunia, the R2R3-MYBs most similar to *AtMYB21* and *AtMYB24* are *NtMYB305* and *EOBII* (see **Supplementary Figure S5**) and are involved in nectary gland formation (Liu et al., 2009) and biosynthesis of phenylpropanoid volatiles (Spitzer-Rimon et al., 2010; Colquhoun et al., 2011), respectively. Similarly, in cotton, *GhMYB24* (R2R3-MYB) is preferentially expressed in anthers/pollen (Li et al., 2013). Its overexpression in *Arabidopsis* leads to sterile plants, while lower to moderate expression levels of transgene produce fertile plants. A similar phenotype was observed when peach *MYB10.1* was overexpressed in tobacco plants, even though the two MYBs belong to different clades. It has to be noted that also peach has a gene of the MYB21/24/57 subclade (see **Supplementary Figure S5**) whose expression in the first three whorls of the flower (**Supplementary Figure S6**) is much higher than those of the previously characterized *MYB10* genes (Rahim et al., 2014), and thus, we could speculate that also in peach flower development is controlled by MYB24, whereas MYB10.1 is controlling anthocyanin synthesis. Nonetheless, the out-titration of MYB proteins achieved by heterologous expression with strong expression systems, such as the use of the Ca MV 35S promoter or a dual system as here, might affect the action of endogenous tobacco MYBs, interfering with functions that they do not (or do marginally) control in the original species.

In transgenic type I tobacco plants overexpressing peach *MYB10.1*, *NtMYB305* showed extremely low levels of expression, similar to those described in NtMYB305 RNAi lines (Liu et al., 2009). Thus, the nectary phenotype of *MYB10.1* overexpressing plant might be explained by the suppression of *NtMYB305*. More complicated is the explanation of the anther phenotype, as *NtMYB305* has been reported to be not expressed in this flower part (Liu et al., 2009), as, on the contrary, is for its strict homologues from *Arabidopsis* (MYB21/MYB24) cotton (Li et al., 2013), but also peach (**Supplementary Figure S6**). Jasmonate (JA) controls several plant processes, including plant growth, fertility, development, anthocyanin accumulation, and defense, and within the signaling cascades activated by JA, the jasmonate-ZIM domain (JAZ) repressor proteins are the major components (reviewed from (Browse, 2009; Pauwels and Goossens, 2011). Thines et al. (2007) demonstrated that AtJAZ1 protein repressed the transcription of JA responsive genes, and application of exogenous JA initiates JAZ1 degradation. Qi et al. (2011) reported that JAZ proteins interact with bHLH TFs like transparent testa8 (TT8), GLABRA3 (GL3), and enhancer of GLABRA3 (EGL3) and R2R3-MYB TFs such as MYB75/PAP1 and GLABRA1 (GL1) to repress JA-mediated anthocyanin production and trichome formation. In the absence of JA, JAZ proteins bind to the downstream TFs and limit their transcriptional activity, while the availability of JA leads to degradation of the JAZ proteins to free the downstream TFs for the transcriptional regulation of target genes (Chini et al., 2007; Qi et al., 2011). Jasmonic acid is involved in stamen development and pollen maturation process in plants. It has been demonstrated (Mandaokar et al., 2006) that *AtMYB21* and *AtMYB24* are induced by JA, while JAZ proteins interact with AtMYB21 and AtMYB24 to decrease their transcriptional function (Song et al., 2011); upon perception of JA signal, COI1 recruits JAZs to the SCF^COI1^ complex for ubiquitination and degradation through the 26S proteasome to release AtMYB21 and AtMYB24, thus triggering transcription of various important genes for JA-mediated stamen development. Moreover, it has been shown that AtMYB21 acts within a negative feedback loop that regulates expression of multiple JA biosynthetic genes and, together with AtMYB24, also affects AtARF6 and AtRF8 activity and that a portion of the *myb21myb24* flower phenotypes may be caused by decreased ARF activity (Reeves et al., 2012). The overexpression of *MYB10.1* also up-regulates the JA biosynthetic and signaling pathway genes *NtAOS* and *NtJAZd* (orthologous to *AtJAZ1* gene), in type I transgenic tobacco flowers. This suggests an imbalance in JA action in these transgenic plants, which show a phenotype similar to JA-deficient mutants but increased levels in transcripts coding for JA synthesis and action. This imbalance occurs only when high levels of *MYB10.1* accumulate in transgenic plants, thus leading to accumulation of flavonoids. It is well known that flavonoids inhibit auxin transport (Peer et al., 2011), and thus the increase in flavonoid concentration might impair auxin actions leading also to reduced JA levels, which could explain the anther phenotype. On the contrary, type II and WT showed a normal expression pattern of JA-related genes, and flowers had normal development, probably because these plants could react to the higher *MYB10.1* levels by decreasing *NtAN2* and *NtMYB305* levels, thus keeping the MYB-bHLH-WD40 (MBW) complex below a threshold level that does not stimulate excessive flavonoid production. Li et al. (2004) characterized a tomato mutant, *jasmonic acid–insensitive1* (*jai1*) with defects in JA signaling resulting in female sterility. They also reported that the sterility was due to the defect in the maternal control of seed maturation linked with the loss of accumulation of JA-regulated proteinase inhibitor proteins in reproductive tissues. Thus, besides auxin, also an impaired JA metabolism might be (among) the cause(s) of altered female fertility also in type I tobacco plants overexpressing *MYB10.1*. Indeed, type I plants exhibit a block during female gametophyte development and in particular during FG1 stage (St3-1), with respect to the WT. The *Arabidopsis* AtARF6 and AtARF8 are expressed also in the ovule and the embryo sac and, with auxin, are crucial for proper gynoecium maturation (Reeves et al., 2012).

The plant hormone auxin is important for gametophytic developmental processes, and it was observed that manipulation of its levels results in changes in cell fate (Panoli et al., 2015). Disruption of such gradients due to flavonoids inhibition of auxin transport might be among the causes of the arrest of the megagametophyte development.

At the same time, ethylene plays an important role in the early stages of female sporogenesis and ovule fertilization in tobacco (De Martinis and Mariani, 1999). Suppression of a pistil-specific *NtACO* gene caused female sterility due to an arrest in ovule development in transgenic tobacco plants. Type I transgenic tobacco flowers showed the opposite result with higher expression level of *NtACO*, possibly to balance the altered JA and IAA levels or because of a feedback mechanism trying to overcome the block of ovule development.

The nectary is rich in carbohydrates and secretes nectar to attract pollinators, as well as defends floral reproductive tissues against microorganisms (Carter et al., 2007). The tobacco nectar contains five different types of nectarin proteins (NEC1 to NEC5), but NEC1 is most abundant, and its expression is restricted to nectary (reviewed from Carter and Thornburg, 2004). The expression of *NtNEC1* is regulated by tobacco *NtMYB305* (Liu et al., 2009). The expression levels of *NtMYB305*, *NtNEC1*, *NtNEC5*, and anthocyanin biosynthetic genes such as *NtPAL* and *NtCHI* were reduced in tobacco by *NtMYB305* knockdown experiment. The type I transgenic flowers also showed similar effects. These transgenic flowers were defective in floral development and had no nectary gland; this could be due to either the extremely low expression of *NtMYB305*, which could not activate transcription of *NtNEC1* in type I transgenic tobacco flowers, or a direct interference of MYB10.1 on *NtNEC1* transcription. Considering these findings, it is hypothesized that the overexpression of *MYB10.1*, by altering IAA/JA/ethylene levels, suppresses *NtMYB305* expression, thus causing defective flowers/missing nectary.

Based on the present results obtained through transgenic plant analysis, it can be concluded that overexpression of *MYB10.1* in tobacco regulates anthocyanin biosynthesis in the reproductive parts. Furthermore, its misregulation interferes, directly or indirectly, in other process such as vegetative and reproductive development, suggesting caution when choosing MYBs and promoters to induce the synthesis of anthocyanins to improve plant nutritional quality (Appelhagen et al., 2018).

## Author Contributions

LT and MR designed the research. MR, FV, and FR conducted the experiments. LT and MR analyzed data. FR, MR, and LT wrote the manuscript. All authors read and approved the manuscript.

## Funding

This research work was supported by Ministero delle Politiche Agricole Alimentari e Forestali-Italy through the project “DRUPOMICS” (grant DM14999/7303/08) and University of Padova (grant CPDA072133/07). MAR was supported by a “Fondazione CARIPARO” fellowship.

## Conflict of Interest

The authors declare that the research was conducted in the absence of any commercial or financial relationships that could be construed as a potential conflict of interest.

## Abbreviations

4-MU, 4-methylumbelliferone; AN2, anthocyanin2; ANR1, anthocyanindin reductase 1; ANS, anthocyanidin synthase; bHLH, basic helix-loop-helix; CHI, chalconeisomerase; CHS, chalcone synthase; DFR, dihydroflavonol 4-reductase; F3H, flavanone-3-hydroxylase; JA, jasmonate; JAZd, jasmonate ZIM-domain d; LAR, leucoanthocyanidin reductase; MTT, 2,5-diphenyl monotetrazolium bromide; MUG, 4-methylumbelliferyl-β-d-glucuronide; NEC1, nectarin1; PAL, phenylalanine ammonia-lyase; PAP1, production of anthocyanin pigment1; qRT-PCR, quantitative real-time polymerase chain reaction; RT-PCR, reverse transcription–polymerase chain reaction; TF, transcription factor; UFGT, UDP-glycose:flavonoid-3-o-glycosyltransferase; WT, wild type.
