# Supplementary material for: Effects on Plant Growth and Reproduction of a Peach R2R3-MYB Transcription Factor Overexpressed in Tobacco
Source: Front Plant Sci. 2019 Oct 18;10:1143. doi: 10.3389/fpls.2019.01143 (PMC6813659; doi:10.3389/fpls.2019.01143)
Supplement: Supplementary file 1 [file Image_1.pdf]

Supplementary Material

Effects on plant growth and reproduction of a peach R2R3-MYB transcription factor overexpressed in tobacco

Md Abdur Rahim[1,2], Francesca Resentini[1,3], Francesca Dalla Vecchia[1] and Livio Trainotti[1]*

[1]Department of Biology, University of Padova, Padova, Italy

[2]Present address: Dept. of Genetics and Plant Breeding, Sher-e-Bangla Agricultural University, Dhaka, Bangladesh

[3]Present address: Instituto de Biología Molecular y Celular de Plantas, Consejo Superior de 20 Investigaciones Científicas (CSIC)—Universidad Politécnica de Valencia, Valencia, Spain

*** Correspondence:**
Livio Trainotti
livio.trainotti@unipd.it

1 Supplementary Tables

Supplementary Table 1. Primers used for gene expression analysis

Gene	GeneBank Accession no.	Forward primers (5´-3´)	Reverse primers (5´-3´)
MYB10.1	ppa026640m*	CAGGAAGGACAGCGAATGATG	TCGGGGTTGAGGTCTTATTACG
MYB10.2	ppa016711m*	ATGGAGGGTTATGACTTGAGTGT	TTACTTTCTATATTCTTCATTTGAAT
MYB10.3	ppa020385m*	ATGGGGGGAAATAACTTGGATGT	TTATTCTTCTTTTGAATGATTCCA
MYB24	ppa011751m*	CAAGTGGGGAAACAGGTGGTC	CTGCTTGCTTAATGTGCTTTTGAA
PpN1	ppa009483m*	CCAGGAGAATCGGTGAGCAGAAAA	TCGAGGGTGGAGGACTTGAGAATG
NtMYB305	EU111679.1	GGACAAGGATTCAGAAGCACATA	GTTGGACCAGCAGACGACATA
NtPAL	D17467.1	CCCCTTCGCGGCACCATCAC	TGCTTTAGAATTAGGCCGACCAGT
NtAN2	FJ472647	GAAGAAAGGTGCATGGACTG	TCTGCAGCTCTTTCTGCATC
NtAN1b	HQ589209	CTTGAACACTTCTCAAACCGA	TGCTAGGGCACAATGTGAAG
NtCHS	AF311783.1	CACGGTCATGGCTATCGGAACA	GCTCAACCTTATGCTCGCTATTA
NtCHI	AB213651	GCCGGCGCAGGAAATAGAGGT	TAGCGGCGAGAAAAGGAAGAGCA
NtF3H	AB289450	CTTACCCTTGGACTGAAACGACAC	CAACGGGCTGAACAGTAATCCA
NtDFR	AB289448	CAGAGAAGGCCGCAATGGAAGC	GGTGGGAATGTAGGCGTGAGGAAT
NtFLS	AB289451	GCTGCGAGAAGTTGTGGAGAAAA	CCTTGGGCATGGTGGGTAATAA
NtLAR	AM827419.1	CTTCAAGGTCCTTTACGCCATC	GCTGCAGAGAATATCAACCCC
NtANR1	AM791704.1	GCAATCTTTGACAGGGAATGAAT	TGGGCGCGACAAACATCTT
NtANS	AB289447	TTAACTACTACCCCAAATGTCC	TGCCGTTACCCACTGTCCTTC
NtUFGT	FG627024.1	CAATGAGTGCATTGGATGCC	CCAGCTCCATTAGGTCCTTG
NtJAZd	JQ172762.1	TTGCGAGACGAAATTCACTTACA	TGCCTTATTTTCCTCATTCTTAGC
NtAOS	AB778304.1	TCGTAGGTGAAGAAGGGGAAAAGT	TCTCGAAACCAGCACCACAAAATC
NtNEC1	AF132671	AAGCCAGGAGCCACAAACAACAAA	TCAATTCGAGCGAGGGATACACCA
NtACO	X98493	CTCGTTGAGAAAGAGGCAGC	GGATCCATCTTGACATCAGA
NtUBC2	AB026056	TGAGAACAAGCGCGAATACAACAG	AACAGATTAAGAGTGCGGGAGATG

* Phytozome accession numbers (www.phytozome.net)

Supplementary Table 2. Comparison of peach MYB10.1 (an R2R3-MYB) TF with anthocyanin promoting *Arabidopsis* and apple R2R3-MYB TFs.

Name of the TFs	GeneBank/TAIR/ Phytozome accessions	gDNA (bp)	CDS (bp)	Protein (aa)	MYB10.1
					Similarity (%)*
**MYB10.1**	Prupe ppa026640m	1858	720	239	100
**AtPAP1/AtMYB75**	AT1G56650/AAG42001	1543	747	248	58.6
**AtPAP2/AtMYB90**	AT1G66390/NP_176813	1585	750	249	63.0
**AtPAP3/AtMYB113**	AT1G66370/NP_176811	971	741	246	52.6
**AtPAP4/AtMYB114**	AT1G66380/NP_176812	1078	420	139	57.7
**MdMYB10**	EU518249.2	4050	732	243	79.2

* CDS for each TFs were compared with MYB10.1 using ClustalW available within the “Lasergene” software package (DNASTAR).

Supplementary Table 3. Pistil fertility test by manual pollination. Twenty flowers for each cross were pollinated and their fertility was assessed as ability to set fruits carrying seeds.

	**♀Type-I (Strong)×♂ WT**	**♀ WT ×♂Type-I (Strong)**
**% fertilized flowers**	0	85

2 Supplementary Figures

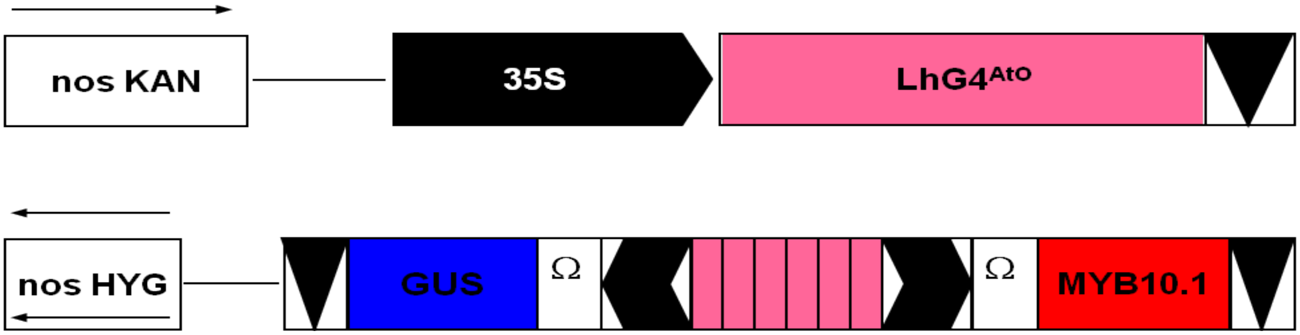

Supplementary Figure 1. Schematic maps of the T-DNA regions of the two binary vectors used for the tobacco transformation. The "*35S::LhG4*" (top) is the LhG4 cassette (activator construct) described in Fig. 1 of (Craft et al., 2005), as it was modified with an improved version of the LhG4 transcriptional activator (i.e. $Gal4^{AtO}$), described by the Moore laboratory in (Rutherford et al., 2005). The "*pOp::MYB10.1*" (bottom) is the pH-TOP-derived cassette (expression construct) described in Fig. 1 of (Craft et al., 2005) in which the peach cDNA encoding the MYB10.1 transcription factor has been cloned, as described previously (Rahim et al., 2014). A different marker gene is present on each cassette to allow the simultaneous selection of both transgenes in co-transformation experiments. The two vectors ($Gal4^{AtO}$ and pH-TOP) were a kind gift of prof. Moore.

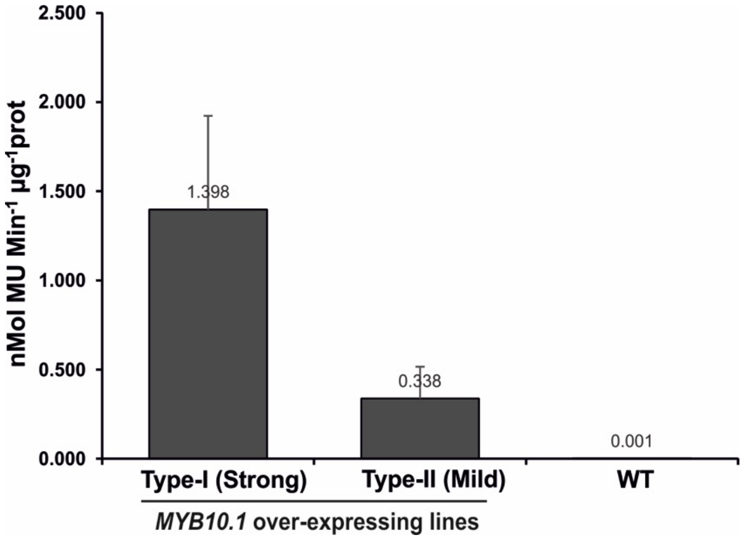

Supplementary Figure 2. Enzymatic GUS activity in MYB10.1 over-expressing transgenic tobacco lines and WT. Error bars are ± SE of the means of three independent plants.

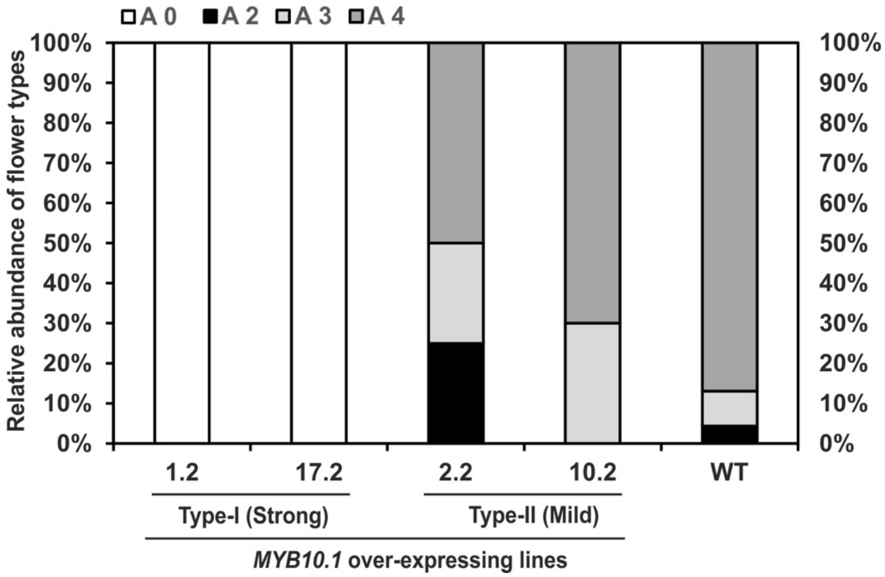

Supplementary Figure 3. The anther filament elongation pattern of *MYB10.1* over-expressing transgenic tobacco flowers. A0, A2, A3 and A4 indicate flowers with 0, 2, 3 and 4 stamens touching the stigmas, respectively.

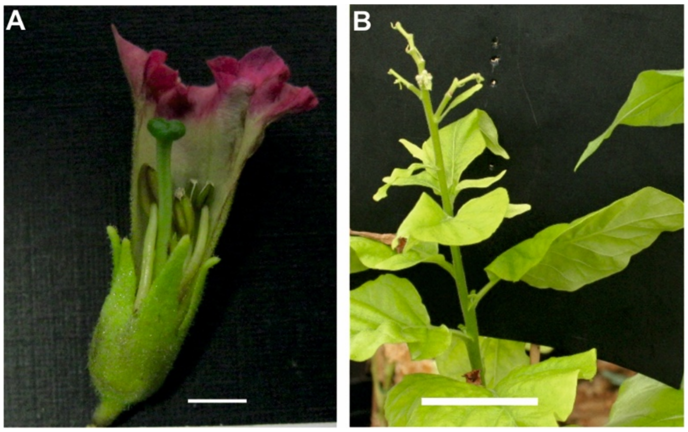

Supplementary Figure 4. Shattering of anthers at extremely late stages of type-I flower development when they are going to abscise (A); due to failure of pollination, fruit setting does not occur and only petioles of the floral inflorescence without capsules remain on type-I transgenic plants (B). Bars are 1 cm (panel A) and 5 cm (panel B).

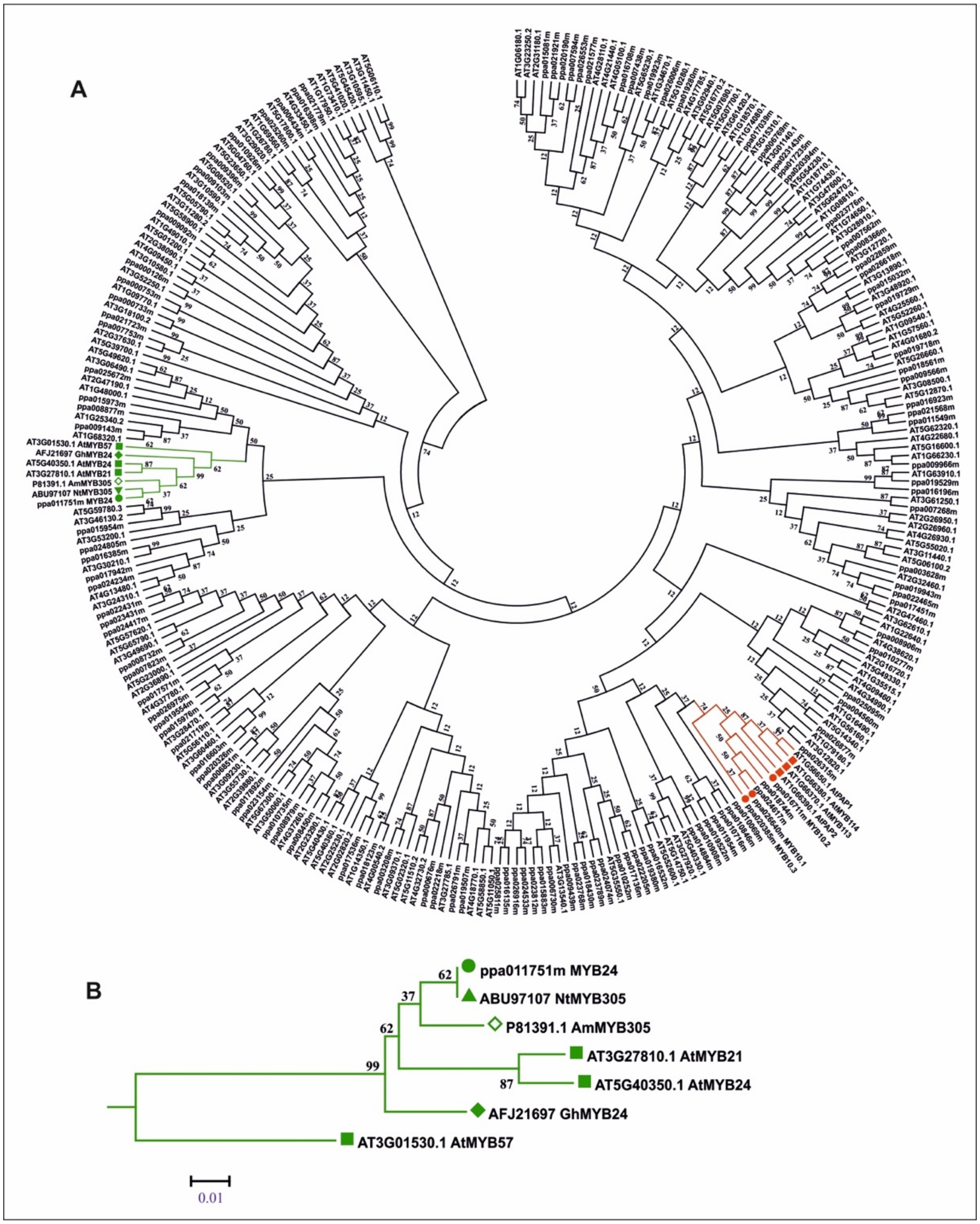

Supplementary Figure 5. Phylogenetic tree showing relationships between *Arabidopsis*, tobacco and peach MYB TFs (A). Magnification of the clade where only MYB TFs related to floral development were clustered (B). Red solid boxes: *Arabidopsis* anthocyanin promoting MYB TFs; green solid boxes: *Arabidopsis* MYB TFs related to flower development (*AtMYB21*, *AtMYB24*, *AtMYB57*); red solid circles: peach MYB10-like TFs; green solid circle: flower specific peach MYB TF, green solid diamond: cotton GhMYB24 (ASJ21697), green unfilled diamond: snapdragon AmMYB305 (P81391) and green solid triangle: tobacco NlxNsMYB305 (ABU97107). The protein sequences of *Arabidopsis* and peach were downloaded from www.phytozome.net. Other protein sequences were retrieved from GeneBank. Phylogenetic and molecular evolutionary analyses were conducted using MEGA version 5.

Description	Mean normalized expression			
	MYB10.1	***M2YB10.2***	***MYB10.3***	***MYB24***
**Complete flower**	**0.290**	**0.143**	**0.038**	**1.876**
**Sepal**	**0.152**	**0.242**	**0.028**	**1.387**
**Petal**	**0.842**	**0.327**	**0.129**	**2.516**
**Androecuim**	**1.354**	**0.195**	**0.160**	**1.783**
**Gynoecium**	**0.006**	**0.022**	**ND**	**0.123**
**Mesocarp around the stone**	**0.593**	**ND**	**0.062**	**ND**

Supplementary Figure 6. Expression levels of *MYB10.1*, *MYB10.2*, *MYB10.3* and *MYB24*, determined by qRT-PCR, in peach flower. RNA from flowers at anthesis (Complete flower) and from their parts (sepals, petals, androecium, and gynoecium) was compared with RNA from previous experiments extracted in fruit mesocarp around the stone (see (Rahim et al., 2014)) for the expression levels of the MYB genes belonging to the AtPAP1/MYB10 clade (known to be activators of the anthocyanin pathway) and MYB24, belonging to the AtMYB21/AtMYB24/NtMYB305, known to regulate flower development. The expression values of the target genes were normalized by the expression values of the *PpN1* gene, used as internal standard. To facilitate the reading and comparison of the expression values, the highest one has been arbitrarily set to 100 (blue), and the others accordingly (0 = white).

The expression levels of the peach *MYB24* (likely orthologous of *NtMYB305*, *AmMYB305*, and *AtMYB21*, *AtMYB24* and *AtMYB57*) and of the tree previously identified *MYB10s* (*MYB10.1*, *MYB10.*2 and *MYB10.1*) genes (Rahim et al., 2014) were analyzed in different parts of the peach flower by qRT-PCR. The expression profiles revealed that in peach, *MYB24* expression was high in sepal, petal, and androecium but low in gynoecium and was undetected in the fruit mesocarp suggesting that *MYB24* is flower specific. In addition, among the three anthocyanin promoting *MYB10* genes, the expression level of *MYB10.1* was the highest, being higher in the androecium followed by petal whereas almost undetectable in the gynoecium. The high transcript level of *MYB10.1* in androecium is in agreement with the anthocyanin content accumulation in purple stamens, highest among other parts of the peach flower.

References

Craft, J., Samalova, M., Baroux, C., Townley, H., Martinez, A., Jepson, I., et al. (2005). New pOp/LhG4 vectors for stringent glucocorticoid-dependent transgene expression in Arabidopsis. *The Plant Journal* 41, 899–918. doi:10.1111/j.1365-313X.2005.02342.x.

Rahim, Md. A., Busatto, N., and Trainotti, L. (2014). Regulation of anthocyanin biosynthesis in peach fruits. *Planta* 240, 913–929. doi:10.1007/s00425-014-2078-2.

Rutherford, S., Brandizzi, F., Townley, H., Craft, J., Wang, Y., Jepson, I., et al. (2005). Improved transcriptional activators and their use in mis-expression traps in Arabidopsis. *The Plant Journal* 43, 769–788.
